# 

**Published:** 1894-03

**Authors:** 


THE Antikamnia Chemical Co., of St. Louis, has issued a visiting list, which is a compact and useful part of a physician's equipment. It has a neat morocco cover with pocket that can be unshipped from the visiting list if desired, or used to protect additional books as they are filled one after another. It is distributed gratis to the profession on application to the publishers.

THE *Woman's Medical Journal*, published in Toledo, Ohio, entered upon its second year in January, and signaled the success of its enterprise by donning a new dress (we had almost said gown), increasing its number of reading pages, and by announcing a new staff of associate editors. This journal appears to be ably edited and managed, and deserves the conspicuous success that it is attaining.

MESSRS. WILLIAM WOOD & Co., New York, announce that the first volume of a new system of Medical Jurisprudence, Forensic Medicine and Toxicology will be published on or about March 1st. In the November issue of the JOURNAL we published a notice in considerable detail of the prospectus of this remarkable work. It will be sold only by subscription, and orders should be placed promptly.

THE McArthur Hypophosphite Co., of Boston, has issued a most useful calendar for the year 1894, of a size convenient for the waistcoat pocket. It contains, among other things, a table for calculating the period of utero-gestation, help in case of accidents, antidotes for poisons, important incompatibles, table of weights and measures and the metric system, valuable information on business matters, doses of chemical and pharmaceutical preparations and a diary arranged for every day in the year. It can be obtained upon application to the publishers and sending twenty cents.

GEORGE KEIL, 1715 Willington street, Philadelphia, announces the early publication (third edition) of the "Medical and Dental Register-Directory and Intelligencer," for the States of Pennsylvania, New York, New Jersey, Maryland and Delaware. It will present not only a complete list of all medical and dental practitioners in the States named, with place and date of graduation, but also lists of professional educational institutions, hospitals, societies, etc., etc., and will be of much practical value to all members of these professions.

---

Frederick Stearns & Co., of Detroit, have sent out a calendar for 1894, that is a unique and beautiful specimen of the application of photography in original colors. The details which attend the process are referred to at length in a circular which accompanies the calendar. The feature of expense in the production of such work is considerable, and while every customer of the firm will receive one of these calendars, duplicates can only be obtained upon payment of twenty-five cents to cover actual cost of production, postage and packing.

---

Civil Service Examinations for Superintendents, First and Junior Assistant Physicians in the State Hospital Service.—An open competitive examination of candidates for positions as Superintendents, and First and Junior Assistant Physicians in the State Hospital service, will be held at the rooms of the New York Civil Service Commission, Albany, N. Y., on Thursday, March 15, 1894, commencing at 9 o'clock A. M.

A candidate for the position of Superintendent must be not less than thirty years of age, and have had five years' actual experience as physician in a hospital for the insane. A candidate for the position of First Assistant Physician must be not less than twenty-five years of age, and have had three years' actual experience in a hospital for the insane. A candidate for the position of Junior Assistant Physician must be at least twenty-one years of age, and have had at least one year's actual experience on the medical staff of a public general hospital. All candidates must be graduates of a legally chartered medical college and citizens and residents of the State of New York.

Application blanks may be had by addressing the Secretary of the New York Civil Service Commission, Albany, N. Y.

THOMAS CARMODY, *Chief Examiner.*

---

Notice to Contributors.—We are glad to receive contributions from every one who knows anything of interest to the profession. Articles designed for publication in the Journal should be handed in before the first day of the month. The Editors are not responsible for the views or opinions of contributors. All communications should be addressed to the Managing Editor, 284 Franklin St., Buffalo, N. Y.

